# Identifying nursing interventions associated with the accuracy used nursing diagnoses for patients with liver cirrhosis ^1^

**DOI:** 10.1590/1518-8345.2016.2933

**Published:** 2017-09-18

**Authors:** Fernanda Raphael Escobar Gimenes, Ana Paula Gobbo Motta, Patrícia Costa dos Santos da Silva, Ana Flora Fogaça Gobbo, Elisabeth Atila, Emilia Campos de Carvalho

**Affiliations:** 2 PhD, Professor, Escola de Enfermagem de Ribeirão Preto, Universidade de São Paulo, PAHO/WHO Collaborating Centre for Nursing Research Development, Ribeirão Preto, SP, Brazil.; 3 Master’s student, Escola de Enfermagem de Ribeirão Preto, Universidade de São Paulo, PAHO/WHO Collaborating Centre for Nursing Research Development, Ribeirão Preto, SP, Brazil. Scholarship holder at Coordenação de Aperfeiçoamento de Pessoal de Nível Superior (CAPES), Brazil.; 4 PhD, Professor, Universidade Federal de Uberlândia, Uberlândia, MG, Brazil.; 5 MSc, Professor, Centro Universitário Módulo, Caraguatatuba, SP, Brazil.; 6 RN, Mona Vale Hospital, Mona Vale, Sydney, NSW, Australia.; 7 PhD, Full Professor, Escola de Enfermagem de Ribeirão Preto, Universidade de São Paulo, PAHO/WHO Collaborating Centre for Nursing Research Development, Ribeirão Preto, SP, Brazil.

**Keywords:** Nursing Care, Nursing Diagnosis, Enteral Nutrition, Liver Cirrhosis, Patient Safety

## Abstract

**Objective:**

to identify the nursing interventions associated with the most accurate and frequently used *NANDA International, Inc.* (NANDA-I) nursing diagnoses for patients with liver cirrhosis.

**Method:**

this is a descriptive, quantitative, cross-sectional study.

**Results:**

a total of 12 nursing diagnoses were evaluated, seven of which showed high accuracy (IVC ≥ 0.8); 70 interventions were identified and 23 (32.86%) were common to more than one diagnosis.

**Conclusion:**

in general, nurses often perform nursing interventions suggested in the NIC for the seven highly accurate nursing diagnoses identified in this study to care patients with liver cirrhosis. Accurate and valid nursing diagnoses guide the selection of appropriate interventions that nurses can perform to enhance patient safety and thus improve patient health outcomes.

## Introduction

Cirrhosis is a chronic degenerative disease characterized by replacement of functional liver tissue by fibrosis. The disease is responsible for high rates of morbidity, mortality, consecutive hospitalizations, work absenteeism, and increases in societal costs. Liver cirrhosis is a public health concern and is the second cause of death amongst gastrointestinal diseases. In Brazil, it is the eighth leading cause of death among men and accounted for almost 9% of hospital admissions in 2010^1^ ^-^ ^2^.

Liver disease affects more people than other types of organ failure. With the progression of the disease, patients can experience associated complications, such as jaundice, portal hypertension, esophageal, gastric and hemorrhoid varices, edema, nutritional deficiency and variceal hemorrhage^3^. Moreover, patients with end-stage liver cirrhosis present calorie and protein malnutrition due to poor intake, absorption, processing and storage of nutrients, resulting in an unfavorable prognosis^4^.

There is no specific cure for cirrhosis. Therefore, the goal of treatment is to minimize the progression of the disease and to prevent complications. In this context, nurses play an important role in the multidisciplinary team because they perform comprehensive and continuous patient care.

To meet comprehensive and complex patient needs in an efficient and safe way, nurses need to have critical thinking skills to accurately diagnose, identify nursing-sensitive patient outcomes and select specific nursing interventions to achieve the desired goals. In patients with liver cirrhosis, nursing care goals may include prevention of complications; promotion, maintenance, and restoration of health; facilitating optimal functional ability in the patients’ desired roles, maximizing well-being, and promoting patient satisfaction^5^.

The use of standardized nursing terminologies in clinical practice contributes to clinical reasoning and decision making to improve healthcare and patient outcomes^6^. The nursing assessment identifies clinical indicators for nursing diagnosis, which represent evidence leading to the identification and implementation of interventions. Accurate and valid nursing diagnoses guide the selection of interventions capable of producing the desired outcomes^7^.

The Nursing Interventions Classification (NIC) figures among the nursing classification systems widely used by nurses around the world^8^. The interventions provided in the NIC facilitate communication among nurses and other healthcare professionals, provide information to administration to balance the cost of components and quality of care, and facilitate the identification of care for specific populations^9^. Although the use of a *standardized nursing language system, including NANDA-I and NIC taxonomies, is well described in the nursing literature, there is a need for further testing in clinical practice to demonstrate their applicability to patient care and to add to a body of evidence in specific patient populations* ^8^.

This study addressed patients with liver cirrhosis as a population because the burden of the disease in Brazil has risen steadily, with ever-increasing associated costs and its effect on hospital admissions and mortality rates^10^. To date, previous studies have shown the effectiveness of nursing interventions in the treatment of nursing diagnoses in diverse populations and in different clinical settings^8^. There has been no specific research though that captures the contribution of nursing interventions to improve care to patients with liver cirrhosis.

## Purpose

The purpose of this study was to identify the nursing interventions associated with the most accurate and frequently used NANDA-I nursing diagnoses for hospitalized patients with liver cirrhosis.

## Methods

In this paper, we present results from a major investigation on nursing diagnosis and interventions in patients with liver cirrhosis^11^. This is a descriptive, quantitative, cross-sectional study, conducted from January 2013 to December 2015.

## Settings and Participants

All nurses working at the gastroenterology ward of a Brazilian university hospital were eligible to participate. The exclusion criteria were nurses on vacation during data collection; participants were all Portuguese speakers; they were between 28 and 62 years of age (mean of 39.8 years) and had been working at the gastroenterology ward between one and 27 years (mean of 8.6 years).

## Ethical Aspects

The study received approval from the Research Ethics Committee of the University of São Paulo at Ribeirão Preto College of Nursing (CAAE: 05759812.4.0000.5393). Researchers provided the participants with oral and written information about the aim and procedure of the research. Participants were assured that their identity would remain confidential and the written consent form stipulated that they could decline or withdraw from the research at any time with no repercussions in their work.

## Procedure

This study was conducted in three phases: (i) evaluating the accuracy of the most frequent used NANDA-I nursing diagnosis for hospitalized patients with liver cirrhosis identified in previous study^11^; (ii) designing of the data collection tool based on the 5^th^ edition of NIC^9^; and (iii) identifying the nursing interventions associated with the most frequent and accurately used NANDA-I nursing diagnosis for these patients. The three phases are explained in the following sections.

### First phase: evaluating the accuracy of the most frequently used NANDA-I nursing diagnosis for patients with liver cirrhosis

An expert panel composed of five nurses reviewed the most frequently used NANDA-I nursing diagnoses identified in a previous study^11^. To evaluate the accuracy of nursing diagnoses, the principal investigator sent the following to the experts: case studies of 20 patients, the Nursing Diagnosis Accuracy Scale (EADE-version 2), adapted to Brazilian culture by Matos and Cruz^12^ from Lunney^13^; a guide for completing the EADE-version 2; and a copy of the 12 most frequently identified nursing diagnoses as stated in a previous study.

The EADE-version 2 allows the nurse to take into consideration the presence, relevance, specificity and coherence of cues to reach a nursing diagnosis. It also indicates, using ordinal values, the degree of diagnostic accuracy into four categories: zero (0), low (1), moderate (2 / 4.5 and 5.5) and upper (9/10 / 12.5 and 13.5). According to Matos and Cruz^12^ cues are determined by the presence or absence of defining characteristics of the nursing diagnosis. Therefore, the expert panel judged if there were clues for each nursing diagnosis formulated by the principal investigator and, in the presence of clues, judged the degree of relevance, specificity and coherence.

The answers given by the expert panel for each item of EADE-version 2 corresponded to a score (High Relevance = 1; High Specificity = 3.5, High Coherence = 8), and the sum of the scores resulted in a final score that indicated the degree of accuracy of the nursing diagnosis. Finally, based on the degree of accuracy obtained, it was possible to identify the accuracy category of each nursing diagnosis (High, Moderate or Nil). A period of 90 days was stipulated for experts to return the analysis to the researcher.

The concordance index (IVC) among experts in the degree of accuracy of nursing diagnoses was calculated as follows: the number of experts who rated the nursing diagnoses as “High Accuracy” was divided by the total number of experts. Researchers considered an IVC equal to or greater than 0.80 as high accuracy^14^.

### Second phase: designing of the data collection tool

The identification of nursing interventions in the NIC was performed only for the nursing diagnoses classified as high accuracy. A data collection tool was developed based on the 5^th^ edition of NIC^9^, as no other instrument was found suitable for this study. The development of this tool occurred in two steps.

In the first step, the principal investigator identified the NIC interventions linked to NANDA-I diagnoses. For each high accuracy diagnosis, researchers identified the list of suggested interventions for resolving the seven identified diagnoses. Then, the principal investigator developed a data collection tool containing three parts. The first part was composed of demographic data of the nurses working in the gastroenterology ward. The second part contained the NANDA-I/NIC linkage, definitions of each nursing intervention chosen from the NIC suggested list^9^, and the assessment scores based on the four-point Likert scale (1 = uncharacteristic; 2 = characteristic; 3 = considerably characteristic; 4 = very characteristic). The purpose was to evaluate how the nursing intervention is used in clinical practice to care for patients with liver cirrhosis.

For the diagnosis Infection, Risk for (00004), a total of 24 interventions and 30 additional optional interventions is suggested in NIC, but researchers elected only 16 of these interventions. Interventions focusing on surgical patients, pregnant women/mothers and newborns were excluded.

For the nursing diagnosis Fluid Volume, Excess (00026), the NANDA-I/NIC linkage suggests a total of 20 interventions and 25 optional interventions. The 18 most comprehensive interventions were selected for patients with liver cirrhosis (e.g. Electrolyte Management [2000] x Electrolyte Management: Hyperkalemia [2002]). Interventions chosen to meet other clinical conditions’ needs (eg. parturient) were excluded.

The NIC interventions linked to the NANDA-I diagnosis Skin Integrity, Risk for Impaired (00047) included a total of 31 interventions and 17 additional optional interventions. Interventions focusing on breastfeeding, plastering or pneumatic tourniquets, surgical patients and latex precautions were excluded, resulting in 17 interventions.

The diagnosis Self-Care Deficit: Bathing (00108) has a total of 14 interventions and 20 additional optional interventions suggested in the NANDA-I/NIC linkage. From those, 12 were selected for cirrhotic patients; those intended for infants and patients with special needs (e.g. dementia) were excluded.

Regarding NANDA-I diagnosis Falls, Risk for (00155), NIC suggests 18 interventions and six additional optional interventions; those interventions for the pediatric age group and patients with special needs were excluded.

For the diagnosis Nutrition: Imbalanced, Less Than Body Requirements (00002), NIC suggests 15 interventions and 24 additional optional interventions. From those, 32 were chosen for patients with liver cirrhosis, and those intended for infants/children were excluded.

Finally, the diagnosis Self-Care Deficit: Dressing (00109) features six interventions suggested by NIC and 12 additional optional interventions; five were selected because they were the most comprehensive (for example, Exercise Promotion [0200] x Exercise Promotion: Stretching [0202]).

The third part of the data collection tool had a field where nurses could describe any comments they deemed necessary, and/or other interventions not suggested in the NANDA-I/NIC linkage which they thought could be useful in clinical practice.

In the second step, the data collection tool was assessed for face and content validity by five experts, all of whom held PhD degrees; were experienced using NIC in teaching, research, or clinical practice; expertise in the nursing process; and who were experienced caring for patient populations with liver cirrhosis. The tool was sent to the experts with an evaluation form attached that contained two parts. The first part consisted of the experts’ identification, and the second part had instructions for completing the data collection tool. The evaluation form included organization, structure, presentation of items, consistency, and formatting of the data collection tool. Each section was evaluated according to four criteria for which the panel members assigned a score from 1 to 4 (1 = *disagree* ; 2 = *partially agree* ; 3 = *agree* ; 4 = *strongly agree*). The evaluation form contained spaces for comments and suggestions about the tool. The experts had 30-60 days to evaluate and return the tool and evaluation form to the researchers. All interventions included in the data collection tool were maintained because they were considered appropriate to care for patients with liver cirrhosis. Thus, data collection tool was considered suitable for application.

### Third phase: identifying the nursing interventions associated with the most frequent and accurately used NANDA-I nursing diagnosis for patients with liver cirrhosis

A total of 10 nurses were working on the ward and one was on leave during the period of data collection, thus nine nurses were enrolled. From those, seven (77.78%) participated voluntarily. Each nurse had 15 days to answer and return the tool in a pre-established schedule. Based on a previous study^15^ and using the data collection tool developed in the first phase, the nurses working in the gastroenterology ward were asked to rate each of the interventions based on the extent to which the intervention was characteristic in their practice with people with liver cirrhosis. The four-point Likert scale (1 = uncharacteristic; 2 = characteristic; 3 = considerably characteristic; 4 = very characteristic) was used for this purpose.

## Data Treatment and Analysis

Data were recorded in a Microsoft Excel^®^ spreadsheet and uploaded to the Statistic Program Package for Social Sciences (SPSS) version 17.0. About the data analysis, the balanced proportions were calculated for each intervention by adding the weights assigned to each response (1 = 0; 2 = 0.33; 3 = 0.67; 4 = 1) and the result divided by the total number of responses. In this study, the nursing interventions with proportions equal to or greater than 0.80 were frequently used by nurses. Interventions with ratios between 0.50 and 0.80 were considered complementary and therefore undertaken occasionally, and interventions with proportions equal to or lower than 0.50 were considered non-essential, or were rarely or never undertaken.

## Findings

In the first phase, the expert panel evaluated a total of 12 labels of nursing diagnoses of NANDA-I and the degree of accuracy. Aspiration, Risk for (00004), Self-Care Deficit: Bathing (00108), Self-Care Deficit: Dressing (00109), Impaired Skin Integrity, Risk for (00047), and Fluid Volume, Excess (00026) were classified as “high accuracy” in 75% or more of patients. For the labels Acute Confusion, Risk for (00173) and Constipation, Risk for (00015), however, three experts (60%) rated them as “high accuracy” in less than 75% of patients. Of the 12 most common diagnosis labels, seven (58.3%) had IVC higher than 0.80, that is, experts agreed that the cues were highly or moderately consistent, relevant and/or specific to the diagnoses in question (Table 1).

**Table 1:** Degree of accuracy of the most frequent nursing diagnoses, according to an expert panel. Ribeirão Preto, SP, Brazil, 2015

**Nursing Diagnosis Labels NANDA-I**	IVC*
**Infection, Risk for (00004)**	**0.99**
**Self-Care Deficit: Dressing (00109)**	**0.95**
**Self-Care Deficit: Bathing (00108)**	**0.93**
**Fluid Volume, Excess (00026)**	**0.87**
**Falls, Risk for (00155)**	**0.87**
**Nutrition: Imbalanced, Less Than Body Requirements (00002)**	**0.83**
**Skin Integrity, Risk for Impaired (00047)**	**0.81**
**Gastrointestinal Motility, Dysfunctional (00196)**	**0.78**
**Bleeding: Risk for (00206)**	**0.76**
**Aspiration, Risk for (00039)**	**0.73**
**Acute Confusion, Risk for (00173)**	**0.67**
**Constipation, Risk for (00015)**	**0.60**

*The bold corresponds to the seven nursing diagnoses labels with IVC (Concordance Index) greater than or equal to 0.8

In the third phase, seven (70%) nurses were asked to rate each of the interventions based on the extent to which the intervention was characteristic in their clinical practice for the care of people with liver cirrhosis for the nursing diagnoses classified as high accuracy. In general, nurses very often performed the chosen interventions, since the average score was greater than 0.8 (Table 2).

**Table 2:** Scores of nursing interventions for high-accuracy nursing diagnoses, according to the participating nurses. Ribeirão Preto, SP, Brazil, 2015

**Nursing Diagnosis Labels NANDA-I**	**Minimum**	**Maximum**	SD*	**Mean**	**n** ^**†**^
**Nutrition: Imbalanced, Less Than Body Requirements (00002)**	**0.62**	**1**	**0.10**	**0.84**	**33**
**Fluid Volume, Excess (00026)**	**0.71**	**1**	**0.09**	**0.88**	**18**
**Skin Integrity, Risk for Impaired (00047)**	**0.76**	**1**	**0.06**	**0.94**	**17**
**Infection, Risk for (00004)**	**0.76**	**1**	**0.08**	**0.93**	**16**
**Self-Care Deficit: Bathing (00108)**	**0.81**	**1**	**0.05**	**0.93**	**12**
**Falls, Risk for (00155)**	**0.62**	**1**	**0.12**	**0.86**	**12**
**Self-Care Deficit: Dressing (00109)**	**0.71**	**0.90**	**0.07**	**0.82**	**6**

*Standard deviation

†Number of interventions recommended for the diagnosis

For the diagnosis Infection, Risk for (00004), one nurse suggested other interventions not provided in the NANDA-I/NIC linkage to solve the problem: Venous Access Devices (VAD) Maintenance (2440) and Skin Care: Topical Treatments (3584) to promote skin hydration.

With regard to the nursing diagnosis Fluid Volume, Excess (00026), the results show that the nurses occasionally used four (22%) interventions to enhance patient outcomes: Medication Management (2380), Weight Management (1260), Neurologic Monitoring (2620), and Urinary Catheterization (0580). One nurse also suggested Skin Care: Topical Treatments (3584) as a useful intervention.

In relation to the NIC interventions linked to the NANDA-I diagnosis Skin Integrity, Risk for Impaired (00047), only one (5.9%) was occasionally performed by nurses: Exercise Therapy: Ambulation (0221). In addition, Infection Control (6540), Wound Care (3660), Bed Rest Care (0740), Circulatory Precautions (4070), and Pressure Ulcer Prevention (3540), were carried out by all nurses participating in the study.

All nursing interventions elected for the diagnose Self-Care Deficit: Bathing (00108) were considered very characteristic in nurses’ clinical practice and one participant suggested “Privacy” as a feasible intervention, although it is not described in NIC.

Regarding NANDA-I diagnosis Falls, Risk for (00155), a total of 12 interventions were selected and two (16.6%) were considered non-essential to treat patients with liver cirrhosis with risk for falls: Exercise Therapy: Muscle Control (0226) and Medication Management (2380). Interestingly one nurse stressed the importance of Physical Restraint (6580).

For the diagnosis Nutrition: Imbalanced, Less Than Body Requirements (00002), the intervention Bowel Management (0430) is frequently used by nurses to meet patient needs. Four interventions (12.5%) were considered non-essential though because they are rarely or never performed by nurses: Eating Disorders Management (1030), Medication Management (2380), Positioning (0840), and Exercise Promotion (0200).

**Figure 1:**
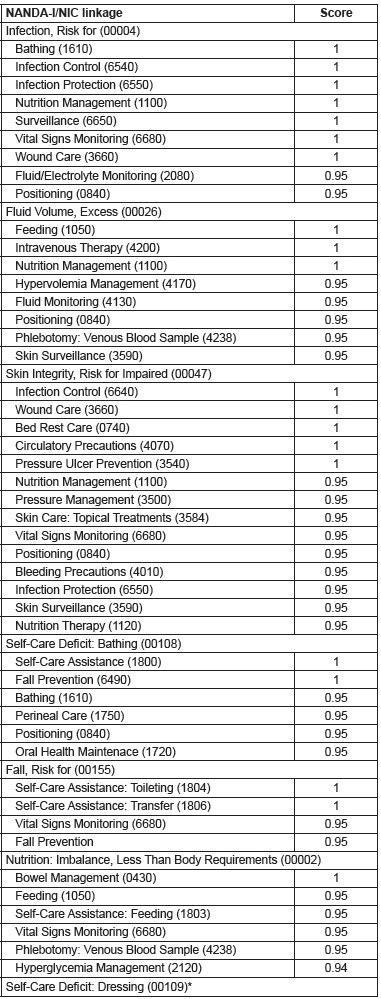
Average scores of NANDA-I/NIC linkage interventions for high-accuracy nursing diagnoses, according to the participating nurses. Ribeirão Preto-SP, Brazil, 2015

From five interventions selected for the diagnosis Self-Care Deficit: Dressing (00109), all were carried out occasionally or very often by nurses to treat patients with liver cirrhosis. According to Figure 1, the following interventions obtained scores superior to 0.90:

*No results greater than 0.90

## Discussion

The purpose of this study was to identify the nursing interventions associated with the most frequent and accurately used NANDA-I nursing diagnoses for hospitalized patients with liver cirrhosis.

The prognosis of patients with advanced cirrhosis is poor because only less than 10% of patients live more than five years. People with decompensated liver cirrhosis integrate a large number of hospitalizations, representing a significant burden for healthcare services^16^. In this context, adequate evaluation and constant monitoring by nurses are strategies that can contribute to the prevention of complications.

In this research, a total of 12 nursing diagnoses were evaluated and seven had high accuracy (IVC ≥ 0.8). It highlights the importance of evaluating and obtaining nursing diagnosis to establish how accurately the information obtained represents the phenomenon and the adequacy of the clinical reasoning process, thus strengthening evidence-based practice^17^.

The nurses reported that they often use the various interventions suggested in NANDA-I/NIC linkage when caring for patients with liver cirrhosis. A total of 70 interventions for high-accuracy nursing diagnoses were identified in this study. From those, 23 were common to more than one diagnosis, including Vital Signs Monitoring (6680); Medication Management (2380); Nutrition Management (1100) and Positioning (0840).

Monitoring vital signs is useful to evaluate patients’ physiological status and the effectiveness of care provided. In a study aimed at identifying nursing interventions in a chemotherapy center, researchers used standardized language systems to find that a greater number of nursing interventions are related to the physiological domain^18^. It is therefore a crucial intervention for patients with chronic conditions, as are interventions focused on medication management using different routes, especially intravenous. Venous access device (VAD) maintenance is another important nursing intervention that enhances patient safety because the activities prescribed may prevent infections and other adverse events.

Additionally, there is concern in the scientific literature regarding nutritional management and the provision of nutritional support that is culturally acceptable, as evidenced in a study conducted in three Czech regions, which determined the frequency of the NIC in terminal patients^19^.

Nurses also suggested other interventions, not described in NANDA-I/NIC linkage, as useful measures in clinical practice to assist patients with liver cirrhosis. These interventions included: VAD Maintenance (2440) and Skin Care: Topical Treatments (3584) to prevent infection; Skin Care: Topical Treatments (3584) to assist patients with excess fluid volume, and the importance of Physical Restraint (6580) to prevent falls.

Nurses also showed concern with the patients’ skin integrity of patients. This organ is the main barrier against infection. Thus, the implementation of specific interventions may decrease the risk of skin breakdown and prevent infection. One possible intervention is to maintain skin integrity by promoting hydration^20^. When performing the nursing process, nurses have a remarkable concern with the diagnoses Infection, Risk and Impaired Skin Integrity, Risk for, and the intervention Skin Care: Topical Treatments (3584); both can be utilized effectively^21^.

In relation to the nursing diagnosis Self-Care Deficit: Bathing, one nurse suggested “Privacy” as a desirable intervention, although it is not described in NIC. Protecting patient privacy is important because nurses have longer direct interactions with patients and therefore expose and manipulate the body when implementing nursing care. For a sick individual, being naked may cause discomfort and embarrassment and the promotion and preservation of patient’s privacy and confidentiality is an essential part of the nurses’ ethical conduct^22^.

Regarding the intervention suggested by one nurse, Physical Restraint (6580) for falls prevention, it should be noted that such interventions are not permitted by law in some European countries, including the UK and the Netherlands^23^. In Brazil, it is an intervention standardized by the Federal Nursing Council and, except in urgent and emergency situations, it is carried out under the direct supervision of a registered nurse and according to established protocols by healthcare institutions^24^. Therefore, there is a worldwide effort to standardize physical restraint techniques aimed at achieving practical methods for use in situations in which it may be necessary for patient protection and safety^25^.

Physical restraint is constantly used in order to reduce falls. The use of this intervention should be assessed by nurses, according to clinical criteria and patient assessment^26^. An alternative approach could be to positively modify the hospital environment to be less hostile and employ better therapeutic restraint management tools; this is a humanizing care task that nurses can foster and advocate within the wider multidisciplinary team^23^.

The researchers concluded that, in general, nurses on one gastroenterology unit characteristically perform nursing interventions that figure among the suggested interventions for the seven most accurate and frequently identified nursing diagnosis in this study to care for patients with liver cirrhosis. Accurate and valid nursing diagnoses guide the selection of appropriate interventions that nurses can perform to enhance patient safety, and thus improve patient health outcomes.

The limitation of this study is that the number of nurses who participated in the third phase of the study was small and limited to one specialty unit in one hospital. It is suggested that the study be conducted with larger samples of nurses, so that interventions that are never performed on patients are identified. Future studies may also identify the reasons why nurses perform some interventions occasionally.

## Conclusion

People with liver cirrhosis are subject to invasive procedures for diagnosis and treatment during hospitalization. It is necessary for nurses to develop skills and competencies in recognizing accurate nursing diagnoses and identifying appropriate nursing interventions in order to provide the best care possible. Accurate and valid nursing diagnoses guide the selection of interventions that improve patient outcomes, thus avoiding rehospitalization due to inadequate nursing care management.
